# Supplementary material for: Two Arabidopsis Splicing Factors, U2AF65a and U2AF65b, Differentially Control Flowering Time by Modulating the Expression or Alternative Splicing of a Subset of FLC Upstream Regulators
Source: Plants (Basel). 2023 Apr 14;12(8):1655. doi: 10.3390/plants12081655 (PMC10145705; doi:10.3390/plants12081655)
Supplement: Supplementary file 1 [file plants-12-01655-s001.zip › plants-2297771-supplementary.pdf]

Supplemental file

Two *Arabidopsis* Splicing Factors, U2AF65a and U2AF65b, Differentially Control Flowering Time by Modulating the Expression or Alternative Splicing of a Subset of *FLC* Upstream Regulators

Hee Tae Lee [1], Hyo-Young Park [1], Keh Chien Lee [1], Jeong Hwan Lee [2] and Jeong-Kook Kim [1,*]

[1] Division of Life Sciences, Korea University, 145 Anam-ro, Seongbuk-gu, Seoul 02841, Republic of Korea

[2] Division of Life Sciences, Jeonbuk National University, 567 Baekje-daero, Deokjin-gu, Jeonju, Jeollabuk-do 54896, Republic of Korea

* Correspondence: jkkim@korea.ac.kr

Received: 7 March 2023; Accepted: 11 April 2023; Published: 14 April 2023

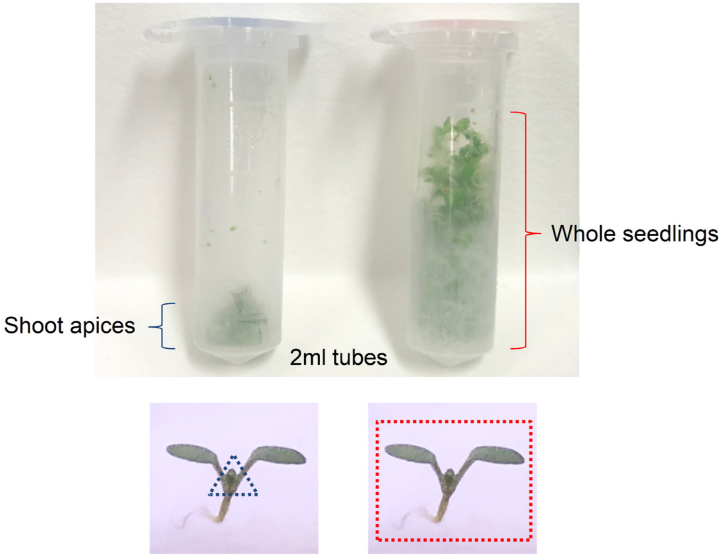

Sample	RNA concentration
Shoot apices (2 plate = 1/6 tube)	4,500 ng/μl
Whole seedlings (1 plate = 2/3 tube)	10,050 ng/μl

Figure S1. Shoot apex regions and whole seedling parts of *Arabidopsis* seedlings used for RNA-seq analysis. The photographs show the shoot apex regions prepared from 7-d-old seedlings by leaf and stem-root cuttings and 7-d-old whole seedlings.

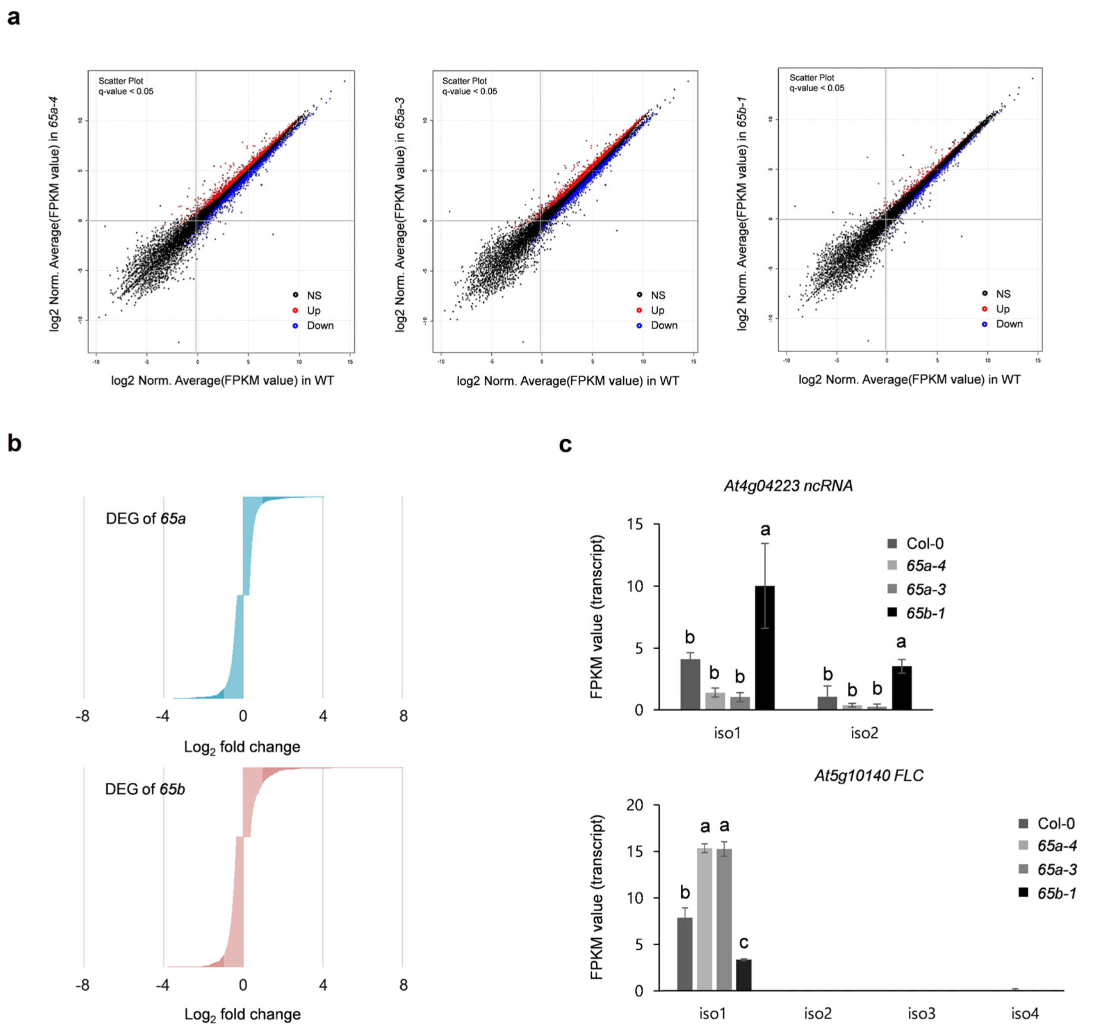

Figure S2. Differentially expressed genes (DEGs) regulated by AtU2AF65a and AtU2AF65b. RNA-seq analysis using shoot apices' RNAs of wild-type (WT, Col-0), *atu2af65a-4*, *atu2af65a-3* and *atu2af65b-1* plants. (**a**) Scatter plots demonstrating DEGs between WT and *atu2af65*-plants. The values of x- and y-axis represent the normalized signal values of WT and mutant samples, respectively. Up, up-regulated; Down, down-regulated; ns, not significant (**b**) DEGs with fold change (FC) in two mutants q-value ≤ 0.05 as the threshold. The DEGs with $|\log_2 FC| \geq 1$ indicate colored dark. (**c**) Transcript levels of *ncRNA* and *FLC* genes with $|\log_2 FC| \geq 1$ based on FPKM values between WT and three *atu2af65* plants. FPKM values of different isoforms for each gene are presented. The letters indicate statistically significant differences ($P < 0.05$) based on Duncan method.

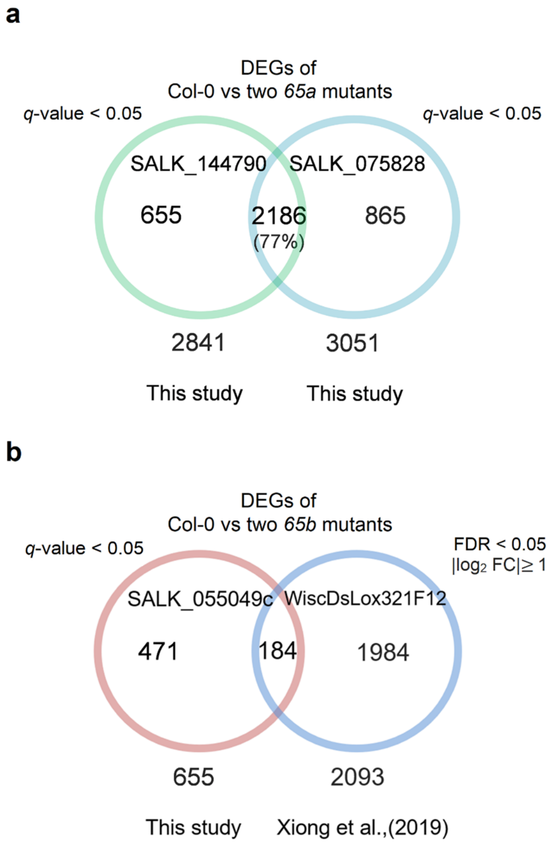

Figure S3. Comparisons of differentially expressed genes regulated by two different mutant alleles of *AtU2AF65a* or *AtU2AF65b*. Venn diagrams showing the numbers of DEGs (**a**) in two different *atu2af65a* mutants (SALK_144790 and SALK_075828) and DEGs (**b**) in two different *atu2af65b* mutants (SALK_055049c and WiscDsLox321F12). Total DEGs of *atu2af65b* (Wisc-DsLox321F12) were from RNA-seq data using 10-d-old shoot apices of Xiong et al., [20].

65a

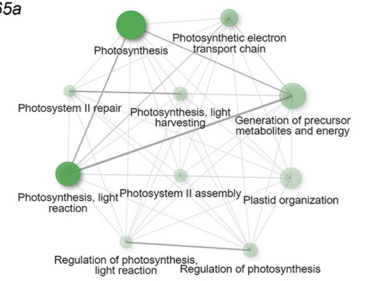

Enrichment FDR	Genes in list	Total genes	Functional Category
$2.9x10^{-43}$	71	275	Photosynthesis
$2.5x10^{-33}$	47	144	Photosynthesis, light reaction
$5.6x10^{-16}$	21	54	Photosynthetic electron transport chain
$6.0x10^{-14}$	55	517	Generation of precursor metabolites and energy
$1.1x10^{-9}$	9	12	Photosystem II repair
$1.5x10^{-9}$	14	41	Regulation of photosynthesis
$3.3x10^{-9}$	13	36	Photosynthesis, light harvesting
$9.3x10^{-9}$	34	310	Plastid organization
$1.3x10^{-8}$	11	26	Photosystem II assembly
$6.3x10^{-8}$	10	23	Regulation of photosynthesis, light reaction

65b

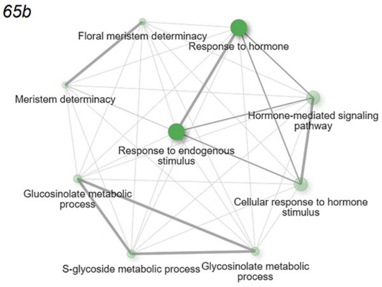

$1.5x10^{-2}$	20	1593	Response to hormone
$1.5x10^{-2}$	20	1631	Response to endogenous stimulus
$1.7x10^{-2}$	14	999	Cellular response to hormone stimulus
$1.7x10^{-2}$	14	944	Hormone-mediated signaling pathway
$1.7x10^{-2}$	5	119	S-glycoside metabolic process
$1.7x10^{-2}$	5	119	Glycosinolate metabolic process
$1.7x10^{-2}$	5	119	Glucosinolate metabolic process
$1.7x10^{-2}$	3	27	Meristem determinacy
$1.7x10^{-2}$	3	23	Floral meristem determinacy

65a&b

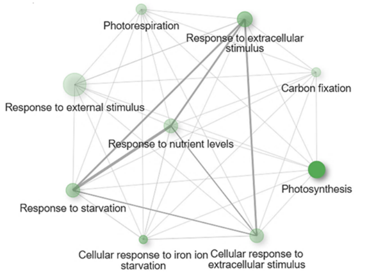

$1.8x10^{-9}$	21	275	Photosynthesis
$2.4x10^{-6}$	17	273	Response to extracellular stimulus
$2.4x10^{-6}$	5	8	Cellular response to iron ion starvation
$8.4x10^{-6}$	14	202	Response to starvation
$1.9x10^{-5}$	14	219	Cellular response to extracellular stimulus
$2.7x10^{-5}$	14	232	Response to nutrient levels
$3.6x10^{-5}$	8	62	Photorespiration
$4.6x10^{-5}$	6	28	Carbon fixation
$7.1x10^{-5}$	44	1914	Response to external stimulus

Figure S4. Gene ontology (GO) enrichment analysis in *atu2af65a* and *atu2af65b* mutants. Total DEGs used in Figure 1b were applied to this GO enrichment analysis. *65a*, DEGs of *atu2af65a* mutants only; *65b*, DEGs of *atu2af65b* mutants only; *65a&b*, overlapped DEGs of *atu2af65a* and *atu2af65b* mutants.

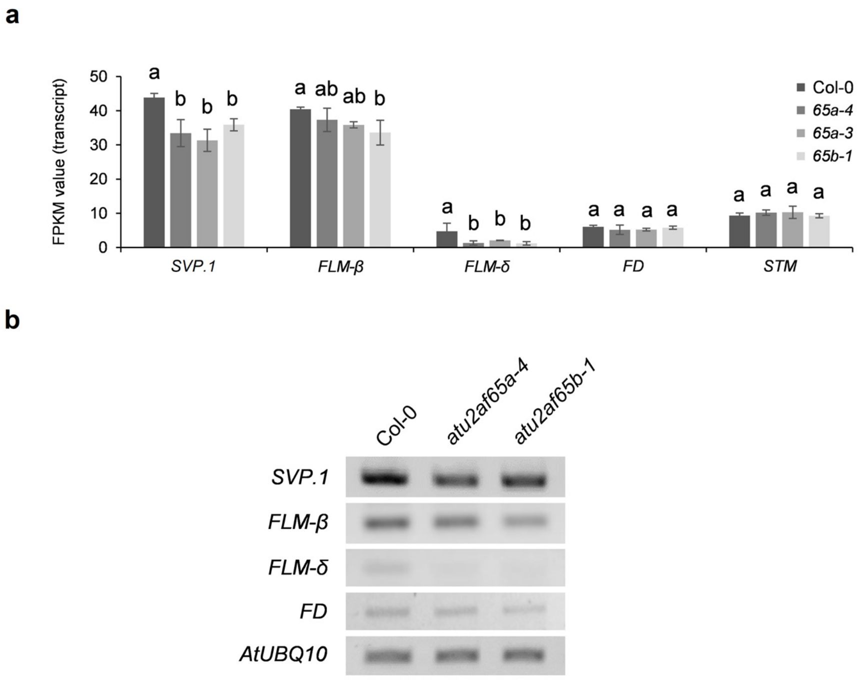

Figure S5. Expression patterns of flowering time genes in shoot apex regions of wild-type (Col-0) and two mutant plants. (**a**) Transcript levels of *SVP.1* (*At2g22540.1*), *FLM-β* (*At1g77080.4*), *FLM-δ* (*At1g77080.2*), and *FD* (*At4g35900.1*) based on FPKM values. *STM* (*At1g62360*) gene was used as a shoot apex specific control. The letters indicate statistically significant differences ($P < 0.05$) based on Duncan method. (**b**) Expression of *SVP.1, FLM-β, FLM-δ,* and *FD* in shoot apices of wild type (Col-0), *atu2af65a-4* and *atu2af65b-1* plants. *AtUBQ10* (*At4g05320*) gene was used as an internal control. It was measured by semi-quantitative RT-PCR analysis.

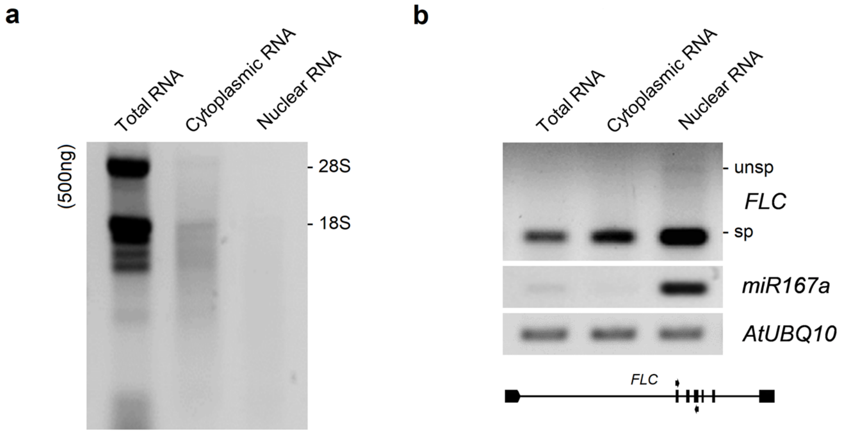

Figure S6. Quality control of nuclear RNA fraction. (**a**) Gel visualization of RNAs of different fractions generated during nuclear RNA purification process. 28S and 18S rRNA bands were much less enriched at nuclear RNA than cytoplasmic RNA in 7-d-old whole seedlings. (**b**) Identification of unspliced *FLC* forms in nuclear RNA fraction. Unspliced *FLC* forms were mostly enriched in nuclear RNA fraction. The primer used for RT-PCR analysis indicated black arrows in *FLC* gene structure. *miR167a* (*At3g22886*) and *AtUBQ10* (*At4g05320*) gene were used as a positive control for nuclear RNA enrichment and an internal control for RT-PCR analysis, respectively.

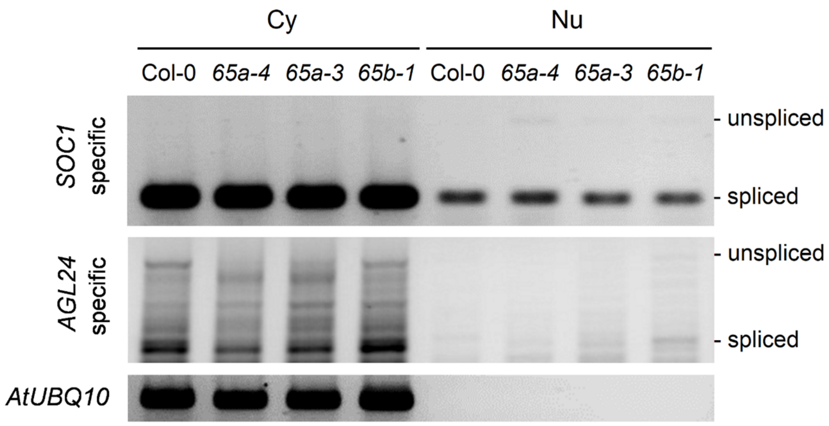

Figure S7. Detection of unspliced and spliced *SOC1* and *AGL24* forms in shoot apices' nuclear fractions of 7-d-old wild-type (Col-0), *atu2af65a-4*, *atu2af65a-3*, and *atu2af65b-1* plants. Cy, cytoplasmic RNA; Nu, nuclear RNA. Note that unspliced RNA was mostly enriched in nuclear RNA fraction, but *SOC1* and *AGL24* splicing defects were not identified in nuclear RNA fraction of *atu2af65a-4* and *atu2af65a-3* mutants.

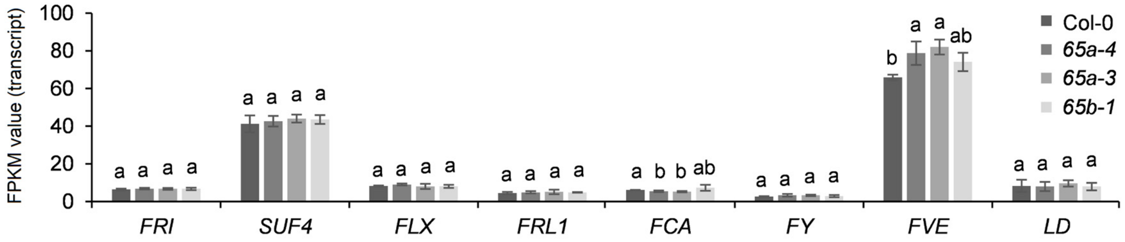

Figure S8. Expression patterns of FRI complex and autonomous genes in shoot apex regions of wild-type (Col-0) and two mutant plants. Transcript levels of *FRI* (*At4g00650.1*), *SUF4* (*At1g30970.1*), *FLX* (*At2g30120.1*), *FRL1* (*At5g16320.1*), *FCA* (*At4g16280.2) FY* (*At5g13480.2*), *FVE* (*At2g19520.1*), and *LD* (*At4g02560.1*) were based on FPKM values. The letters indicate statistically significant differences ($P < 0.05$) based on Duncan method.

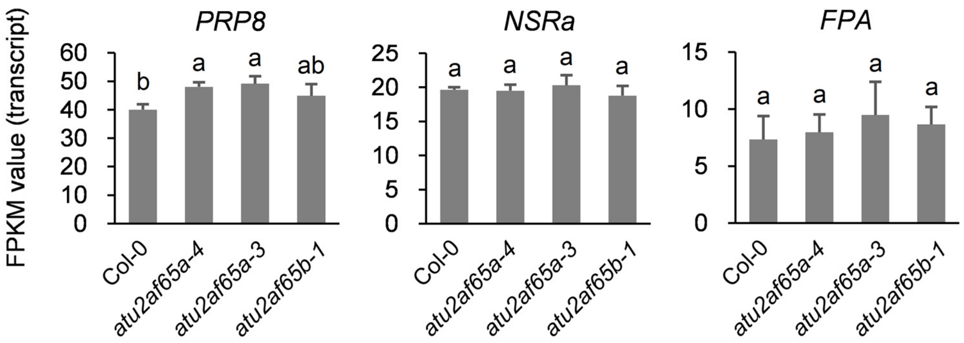

Figure S9. Expression patterns of *PRP8* (*At1g80070.1*), *NSRa* (*At1g76940.1*) and *FPA* (*At2g43410.2*) based on FPKM values. The letters indicated statistically significant differences ($P < 0.05$) based on Duncan method.

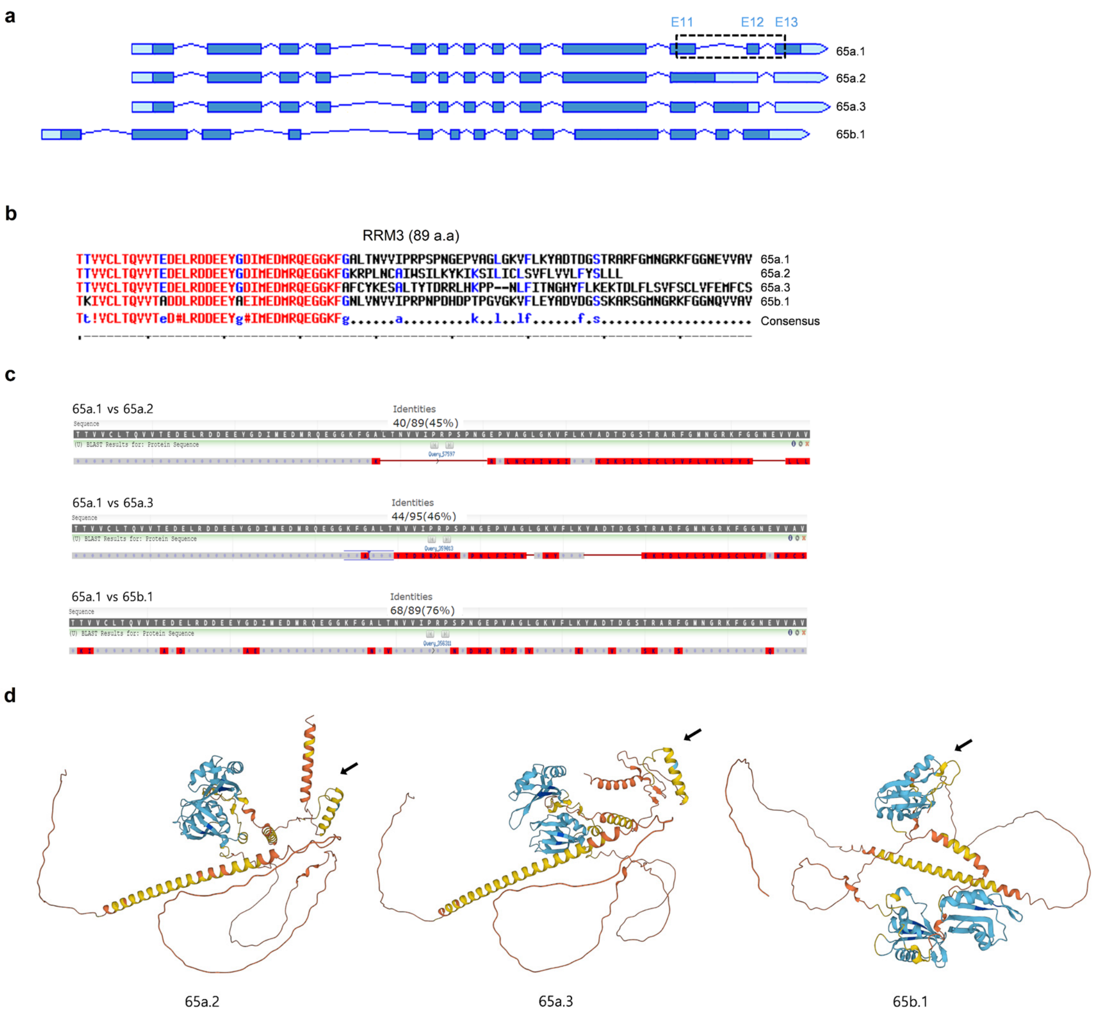

Figure S10. Comparison between AtU2AF65a Isoform 1, 2, 3 and AtU2AF65b. (**a**) Gene structure showing the AtU2AF65a Isoform 1 (65a.1), AtU2AF65a Isoform 2 (65a.2), AtU2AF65a Isoform 3 (65a.3) and AtU2AF65b (65b.1) from The Arabidopsis Information Resource (TAIR, https://www.arabidopsis.org/index.jsp). The dotted line box indicate 65a.1 RNA recognition motif 3 (RRM3) region. (**b**) Multiple sequence alignment of RRM3 (89 amino acid) from multalin (http://multalin.toulouse.inra.fr/multalin/). (**c**) Protein blast of 65a.2, 65a.3, 65b.1 compared to 65a.1 from Needleman-Wunsch Global Align Protein Sequences (Global Align, https://blast.ncbi.nlm.nih.gov/Blast.cgi). (**d**) Protein 3D structure of 65a.2, 65a.3 and 65b.1 were predicted at AlphaFold Protein Structure Database (https://alphafold.ebi.ac.uk/). The black arrows indicate RRM3 region.

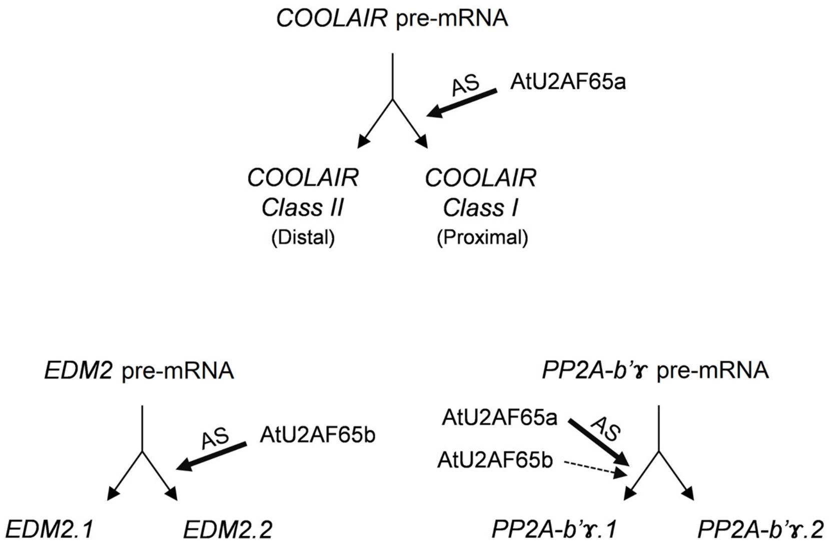

Figure S11. Proposed model for the roles of AtU2AF65a and AtU2AF65b in the alternative splicing of *COOLAIR*, *EDM2*, and *PP2A-b'ɤ* pre-mRNAs. AS, alternative splicing.

Table S1. Summary of reads per each sample and replicate

No	Type	WT	*atu2af65a-4*	*atu2af65a-3*	*atu2af65b-1*
Rep_1	Raw data reads	47,065,642	45,464,656	58,596,272	55,537,190
	Clean reads	44,466,774 (94.5%)	42,770,976 (94.1%)	54,792,960 (93.5%)	52,456,824 (94.5%)
	Mapped reads	43,092,549 (96.9%)	41,260,114 (96.5%)	53,209,151 (97.1%)	50,880,716 (97.0%)
	Uniquely mapped reads	41,622,369 (93.6%)	41,622,369 (93.6%)	51,867,471 (94.7%)	49,638,813 (94.6%)
	Splice	17,401,960 (39.1%)	17,089,369 (40.0%)	22,366,781 (40.8%)	21,120,631 (40.3%)
Rep_2	Raw data reads	41,105,988	51,275,834	44,808,102	48,428,988
	Clean reads	37,917,968 (92.2%)	48,255,618 (94.1%)	42,624,958 (95.1%)	45,612,050 (94.2%)
	Mapped reads	36,662,742 (96.7%)	46,799,204 (97.0%)	41,348,484 (97.0%)	44,216,016 (96.9%)
	Uniquely mapped reads	35,599,947 (93.9%)	45,456,224 (94.2%)	39,921,227 (93.7%)	42,845,594 (93.9%)
	Splice	15,312,389 (40.4%)	19,447,605 (40.3%)	17,376,562 (40.8%)	18,740,092 (41.1%)
Rep_3	Raw data reads	42,919,582	44,926,442	45,003,956	47,139,608
	Clean reads	40,309,730 (93.9%)	42,794,944 (95.3%)	42,449,996 (94.3%)	45,000,960 (95.5%)
	Mapped reads	38,967,698 (96.7%)	41,497,190 (97.0%)	41,161,367 (97.0%)	43,633,622 (97.0%)
	Uniquely mapped reads	37,283,545 (92.5%)	40,162,798 (93.8%)	39,591,742 (93.3%)	41,827,606 (92.9%)
	Splice	15,638,533 (38.8%)	17,435,386 (40.7%)	17,194,658 (40.5%)	17,927,630 (39.8%)

Table S2. List of misregulated floral activator genes in *atu2af65a* and *atu2af65b* mutants

DEG	Gene	Symbol	Log_2 fc 65a	Log_2 fc 65b	Conditions for effect	Appears in
up 65a	AT1G04870	PRMT10	0.37		SD and LD	General processes & autonomous pathway
	AT3G04610	FLK	0.32		SD and LD	General processes & autonomous pathway
	AT3G59060	PIF5, PIL6	0.55		MD (12h light/ 12h dark)	Temperature pathway
	AT4G31120	SKB1, PRMT5	0.42		SD and LD	General processes & autonomous pathway
	AT5G40490	HLP1	0.46		SD and LD	Vernalization, General processes & autonomous pathway
up 65a&65b	-	-			-	-
up 65b	-	-			-	-
down 65a	AT1G26260	CIB5	-0.57		LD [no data under SD]	Temperature pathway, FLC regulation through protein complexes, General processes & autonomous pathway
	AT1G51140	FBH3, BHLH122	-0.33		SD and LD	Photoperiod pathway, Time-course regulation of photoperiodic pathway
	AT2G45660	SOC1	-0.43		SD and LD	Temperature pathway, Hormone pathway, Aging pathway, Photoperiod pathway, Sugar pathway, Mains targets of ABC(E)genes, Specification of flower meristems, Vernalization
	AT4G24540	AGL24	-0.83		SD and LD	General processes & autonomous pathway
	AT5G15840	CO	-0.67		LD only	Circadian clock, Photoperiod pathway, Time-course regulation of photoperiodic pathway
	AT5G47640	NF-YB2, HAP3B	-0.55		LD only	Hormone pathway, Photoperiod pathway, Time-course regulation of photoperiodic pathway
down 65a&65b	AT1G68050	FKF1, ADO3	-0.75	-0.76	SD only	Circadian clock overview, Photoperiod pathway, Time-course regulation of photoperiodic pathway
down 65b	-	-			-	-
reverse	-	-			-	-
			-0.9	0.9		

Table S3. List of misregulated floral repressor genes in *atu2af65a* and *atu2af65b* mutants

DEG	Gene	Symbol	Log_2 fc 65a	Log_2 fc 65b	Conditions for effect	Appears in
up 65a	AT2G38810	HTA8	0.36		SD and LD	General processes & autonomous pathway
	AT4G36920	AP2	0.35		SD and LD	Aging pathway, Photoperiod pathway, Time-course regulation of photoperiodic pathway, Mains targets of ABC(E)genes, Kinetics of ABC(E) genes expression
up 65a&65b	AT2G27550	ATC	1.43	1.11	SD and LD	Photoperiod pathway
	AT5G39550	VIM3, ORTH1	0.47	0.46	LD [no data under SD]	General processes & autonomous pathway
	AT5G65070	MAF4, AGL69	1.04	1.72	SD and LD	FLC regulation during a cold period
	AT5G65080	MAF5, AGL68	4.07	4.41	SD only	FLC regulation during a cold period
up 65b	AT1G01060	LHY		0.59	SD only	Circadian clock
down 65a	AT2G22540	SVP	-0.41		SD and LD	Temperature pathway, Aging pathway, Photoperiod pathway, Mains targets of ABC(E)genes, Specification of flower meristems, Vernalization
	AT2G25930	ELF3	-0.40		SD and LD	General processes & autonomous pathway
	AT3G04030	MYR2	-0.86		SD and LD	Photoperiod pathway
	AT3G15354	SPA3	-0.39		SD only	Photoperiod pathway
	AT5G18240	MYR1	-0.71		SD and LD	Photoperiod pathway
	AT5G37260	RVE2, CIR1	-0.39		LD [no data under SD]	none
down 65a&65b	AT2G40080	ELF4	-0.56	-0.84	SD and LD	Temperature pathway, Circadian clock overview, Photoperiod pathway
	AT3G20810	JMJ30	-0.64	-0.51	SD and LD	Temperature pathway, Time-course regulation of photoperiodic pathway, General processes & autonomous pathway
down 65b	AT1G68840	TEM2		-0.47	LD [no data under SD]	Temperature pathway, Hormone pathway, Photoperiod pathway
	AT3G54990	SMZ		-0.50	SD and LD	Temperature pathway, Hormone pathway, Aging pathway, Photoperiod pathway, Time-course regulation of photoperiodic pathway, Vernalization
	AT5G65050	MAF2, AGL31		-0.38	SD and LD	Temperature pathway, FLC regulation during a cold period
	AT5G65060	MAF3, AGL70		-0.50	SD and LD	FLC regulation during a cold period
reverse	AT5G10140	FLC	1.04	-1.16	SD and LD	Photoperiod pathway, Vernalization, FLC regulation during a cold period
			-4.5	4.5		

Table S4. List of the genes from comparison between transcript DEGs and rMAT in *atu2af65a* and *atu2af65b* mutants

Overlap between 65a transcript DEGs vs 65a rMAT			
No	Gene Access	Gene	AS type
1	AT1G02850	BGLU11	A3SS, RI
2	AT1G03457	-	A3SS
3	AT1G06960	-	A3SS
4	AT1G09140*	SR30	A3SS, RI
5	AT1G12250	-	SE, A5SS
6	AT1G13860	QUL1	A3SS
7	AT1G23020	FRO3	A3SS
8	AT1G28330	DYL1	SE
9	AT1G28395	-	A3SS, RI
10	AT1G65270	-	A3SS
11	AT1G73470*	-	RI
12	AT1G73650	-	RI
13	AT2G28290	SYD	A3SS
14	AT2G30170	-	RI
15	AT2G36670	-	SE
16	AT2G41720	EMB2654	RI
17	AT2G44920	-	RI
18	AT3G16230	-	SE
19	AT3G48050	-	RI
20	AT3G61010	-	RI
21	AT4G02260	RSH1	SE
22	AT4G02640	BZO2H1	A3SS
23	AT4G12560	CPR30	A5SS
24	AT4G16990	RLM3	SE
25	AT4G19550	-	SE
26	AT4G22540	ORP2A	A3SS
27	AT4G26650	-	SE
28	AT4G32850	nPAP	A3SS, RI
29	AT5G04430	BTR1	A3SS
30	AT5G13800	PPH	SE
31	AT5G19030	-	A3SS
32	AT5G22620	-	SE
33	AT5G36170	HCF109	A3SS
34	AT5G48150	PAT1	A3SS
35	AT5G50330	-	SE
36	AT5G53050	-	A5SS
37	AT5G61310	-	A3SS

Overlap between 65b transcript DEGs vs 65b rMAT			
No	Gene Access	Gene	AS type
1	AT1G10657	-	A3SS
2	AT1G22750	-	A3SS
3	AT1G28395	-	SE, A3SS
4	AT1G51200	SAP2	SE
5	AT1G73650	-	A3SS, RI
6	AT2G29630	PY	RI
7	AT2G36670	-	SE
8	AT3G61010	-	RI
9	AT4G00560*	-	SE, A3SS
10	AT4G02260	RSH1	SE
11	AT4G30200	VIL2	A3SS
12	AT4G36690*	AtU2AF65a	A3SS, RI
13	AT5G25520	-	SE, A3SS, RI
14	AT5G37850*	SOS4	A5SS
15	AT5G60170	-	A3SS

* The splicing patterns of these genes were further analyzed in this study.

Table S5. Oligonucleotides used in this study

Direction	No	Gene	Primers (5' to 3')
	1	FLC	AACAGCATGCTGATGATCTTAAAG
	2	SOC1	GGATCGAGTCAGCACCAAACC
	3	AGL24	TTCTCTCTCACTCACACATTAGG
	4	SOC1	AGCCAATCGGGAAATTAACTAAAGA
	5	SOC1	TTTATGTGTGTGACTAATCTTGTTATTG
	6	AGL24	TCCAGCTCAAGAATGAGAGACAT
	7	AGL24	TTTATAAGAAAGATGGATTGTTTGTTGAAA
	8	COOLAIR	CTCGATGCAATTCTCACACG
	9	FLC	CTTAAAGCCTTGGATCATCAGTCA
	10	IPP2	GTATGAGTTGCTTCTCCAGCAAAG
	11	EDM2	TTCCAGCCTGAGATTTCTGTTCT
	12	PP2A-b′ɣ	CCATTTGGCTGTGTTCAATGATTA
	13	EDM2	GTCATAGTATAATCTCCATCAACACA
	14	PP2A-b′ɣ	TTGATTTCATTCATCTCCAGTGCC
	15	SR30	AAGCTGTAGCTATAGTAGCAAGAGCAGGAG
Forward (F)	16	NA	AGTGACTGATTCAGATGACCAGTGT
	17	NA	ATCAACGTACCTACTTCTCTTGTAAACTGG
	18	SOS4	GTTAAAAAGTCTCATGGGTCAAACAGAAG
	19	AtU2AF65a	AGGGAACAATGCTACAAAAGCCT
	20	AtU2AF65a	GAAGGCGGAAAGTTTGGTGCTT
	21	AtU2AF65a	AAGTTTGCCTTTTGCTATAA
	22	PP2AA3	GCGGTTGTGGAGAACATGATACG
	23	AtU2AF65b	CCGAGCAAGAAGAAGTCTTACT
	24	SVP	CTGTAGCTCCAGCATGAAGGAA
	25	FLM	AACTTAGAGCCTTAGATCTTGAAGAAA
	26	FLM	CCGGTGACGAGATAGAAGCG
	27	FD	TAATCTTCATACCCACCATCAC
	28	miR167a	GAAGCTGCCAGCATGATCTA
	29	SOC1	ACCTATACATACACAAACCCTTTATC
	30	AGL24	TTCTCTCTCACTCACACATTAGG
	31	AtUBQ10	CACCATTGACAACGTGAAGG
	1	FLC	GACATCTCCATCTCAGCTTCTGC
	2	SOC1	CCCAATGAACAATTGCGTCTC
	3	AGL24	TGTTATGTTATCAATCTTCTTTATCCTTA
	4	SOC1	CTTTAGGAGAGAAGATGATAAGAGA
	5	SOC1	CATTCTCTATTCTCTTCATCTGAGTT
	6	AGL24	CCGGAGATGAGTAGAAGGTG
	7	COOLAIR	TCCTTGGATAGAAGACAAAAAGAGA
	8	COOLAIR	TATCTTCTGTTTTGTGCTCTTTTACTTT
	9	COOLAIR	TTCTCCTCCGGCGATAAGTA
	10	FLC	TGAACAAGAGCATCGATACTCACA
	11	IPP2	GAGGATGGCTGCAACAAGTGT
	12	EDM2	TAAGAAACTGAATGGAATGCAACGT
	13	PP2A-b′ɣ	CTACTTCGCAACAAGCCTAAACTA
	14	EDM2	CGTTATCAGATCTTCCTCGCAA
	15	PP2A-b′ɣ	GTTTGGATTCGAGTCATTATGCG
Reverse (R)	16	SR30	TGGCCAGTTTTCATTTTCAACCAGATATC
	17	NA	AAGGATTTTCTCAAGCTCATATAACTCGG
	18	NA	ACCATTTGAGCCATTTGAACACGAGAT
	19	SOS4	ATACCCCAACAATTGAAGAGGAAAGACA

20	AtU2AF65a	AATCACCCTGTTCAAACTTGTCTT
21	AtU2AF65a	CCATCTGTATCGGCATACTTCAA
22	AtU2AF65a	TAAGAAATAGTGGCCATTG
23	PP2AA3	GAACCAAACACAATTCGTTGCTG
24	AtU2AF65b	CATACTTGTCTTCGGGGTAATAC
25	SVP	TGAAGTTCGCTGATCTCACTCATA
26	FLM	TCTTTCCCATCTGGCTAGCCA
27	FLM	TCTTCAAGCTTGCTTTGGACTGTTT
28	FD	CAATCCCCAAAAGAGAAACAAG
29	miR167a	ACATAAATTCTTCAGATCCACATAC
30	SOC1	CATTCTCTATTCTCTTCATCTGAGTT
31	AGL24	CCGGAGATGAGTAGAAGGTG
32	AtUBQ10	ACGCAGGACCAAGTGAAGAG

Table S6. Oligonucleotides used in this study

Usage Data	Combination	Gene Access	Gene Detail	Purpose
Fig. 2c	F1 + R1	AT5G10140	FLC	RT-PCR
	F2 + R2	AT2G45660	SOC1	RT-PCR
	F3 + R3	AT4G24540	AGL24	RT-PCR
Fig. 2d	R4	AT2G45660	SOC1 specific	Oligo of RT
	F4 + R5	AT2G45660	spliced SOC1	qPCR
	F5 + R5	AT2G45660	unspliced SOC1	qPCR
	R6	AT4G24540	AGL24 specific	Oligo of RT
	F6 + R6	AT4G24540	spliced AGL24	qPCR
	F7 + R6	AT4G24540	unspliced AGL24	qPCR
Fig. 4b-d	F8 + R7	AT5G01675	COOLAIR Class I	qPCR
	F8 + R7	AT5G01675	COOLAIR Class I	RT-PCR
	F8 + R8	AT5G01675	COOLAIR Class II	qPCR
	F8 + R9	AT5G01675	COOLAIR Class II	RT-PCR
	F9 + R10	AT5G10140	FLC	qPCR
	F1 + R1	AT5G10140	FLC	RT-PCR
	F10 + R11	AT3G02780	IPP2	qPCR
Fig. 5a-b	F11 +R12	AT5G55390	EDM2	qPCR
	F12 + R13	AT4G15415	PP2A-b′γ.1	qPCR
	F10 + R11	AT3G02780	IPP2	qPCR
Fig. 5c	F13 +R14	AT5G55390	EDM2	RT-PCR
	F14 + R15	AT4G15415	PP2A-b′γ	RT-PCR
Fig. 6d	F15 + R16	AT1G09140	SR30	RT-PCR
	F16 + R17	AT1G73470	NA	RT-PCR
Fig. 6e	F17 + R18	AT4G00560	NA	RT-PCR
	F18 + R19	AT5G37850	SOS4	RT-PCR
	F19 + R20	AT4G36690	AtU2AF65a	RT-PCR
Fig. 7a	F20 + R21	AT4G36690	AtU2AF65a.1	qPCR
	F21 + R22	AT4G36690	AtU2AF65a.3	qPCR
	F22 + R23	AT1G13320	PP2AA3	qPCR
Fig. 7b	F19 + R20	AT4G36690	AtU2AF65a	RT-PCR
Fig. 7c	F23 + R24	AT1G60900	AtU2AF65b	RT-PCR
Fig. S5b	F24 + R25	AT2G22540	SVP.1	RT-PCR
	F25 + R26	AT1G77080	FLM-β	RT-PCR
	F26 + R27	AT1G77080	FLM-δ	RT-PCR
	F27 + R28	AT4G35900	FD	RT-PCR
Fig. S6b	F1 + R10	AT5G10140	FLC	RT-PCR
	F28 + R29	AT3G22886	miR167a	RT-PCR
Fig. S7	R4	AT2G45660	SOC1 specific	Oligo of RT
	F29 + R30	AT2G45660	SOC1	RT-PCR
	R6	AT4G24540	AGL24 specific	Oligo of RT
	F30 + R31	AT4G24540	AGL24	RT-PCR
	F31 + R32	AT4G05320	AtUBQ10	RT-PCR
